# Determination of Dominant Frequency of Resting-State Brain Interaction within One Functional System

**DOI:** 10.1371/journal.pone.0051584

**Published:** 2012-12-17

**Authors:** Yu-Jin Zhang, Lian Duan, Han Zhang, Bharat B. Biswal, Chun-Ming Lu, Chao-Zhe Zhu

**Affiliations:** 1 State Key Laboratory of Cognitive Neuroscience and Learning, Beijing Normal University, Beijing, People’s Republic of China; 2 Departments of Radiology, University of Medicine and Dentistry of New Jersey, Newark, New Jersey, United States of America; National Research & Technology Council, Argentina

## Abstract

Accumulating evidence has revealed that the resting-state functional connectivity (RSFC) is frequency specific and functional system dependent. Determination of dominant frequency of RSFC (RSFC_df_) within a functional system, therefore, is of importance for further understanding the brain interaction and accurately assessing the RSFC within the system. Given the unique advantages over other imaging techniques, functional near-infrared spectroscopy (fNIRS) holds distinct merits for RSFC_df_ determination. However, an obstacle that hinders fNIRS from potential RSFC_df_ investigation is the interference of various global noises in fNIRS data which could bring spurious connectivity at the frequencies unrelated to spontaneous neural activity. In this study, we first quantitatively evaluated the interferences of multiple systemic physiological noises and the motion artifact by using simulated data. We then proposed a functional system dependent and frequency specific analysis method to solve the problem by introducing anatomical priori information on the functional system of interest. Both the simulated and real resting-state fNIRS experiments showed that the proposed method outperforms the traditional one by effectively eliminating the negative effects of the global noises and significantly improving the accuracy of the RSFC_df_ estimation. The present study thus provides an effective approach to RSFC_df_ determination for its further potential applications in basic and clinical neurosciences.

## Introduction

Accumulating evidence has demonstrated that the frequency-specific synchrony of cerebral activities in the absence of external stimuli (i.e. resting-state functional connectivity, RSFC) plays an important role in supporting neural communication and brain functional integration [1], [2]. With electrophysiological techniques, the synchronization of neuronal activity has been widely observed. The neuronal electrical signals have been generally classified into several rhythm components which cover frequencies approximately from 0.01 to 500 Hz [2], [3], [4]. The amplitude, or power, of these frequency-specific rhythms is strongly associated with the extent of local synchronization in large neuron populations [5]. The power increase (i.e., synchronization) or decrease (i.e., de-synchronization) in a specific rhythm has been noted to have a specified functional significance. For instance, gamma rhythm (30–100 Hz) is referred to be the mechanism which would account for perceptual binding [3]. And alpha rhythm (∼10 Hz) is considered to be a neural baseline with “inattention” [5]. Coinciding with the local synchronization, the long-distance synchronization, measured by the coherence between rhythms from different recordings sites, also manifests a frequency specific and functional dependent characteristic. For example, the synchronization between temporal and parietal cortex evolves in the lower beta frequency band (12–18 Hz) during multimodal semantic processing; the synchronization between frontal and parietal cortex in the theta (4–8 Hz) and alpha frequency band associated with processing of internal mental context or top-down processing [6]. Taking advantage of brain imaging techniques with fine spatial resolution (e.g. fMRI), study goes further into the dominant frequency of the RSFC (RSFC_df_) within specific brain functional systems. Literature has revealed that RSFC_df_ tends to show a difference between various functional systems. For example, the connectivity in the sensorimotor systems was found to be predominant at a low frequency band, usually below 0.1 Hz (0–0.1 Hz in [7], 0.01–0.06 Hz in [8], and 0–0.08 Hz in [9]). The dominant frequency band of the connectivity between bilateral amygdala, however, was relatively broader and higher (0–0.14 Hz in [8] and 0–0.25 Hz in [9]). The neurophysiological basis of this functional system dependence has been investigated by the studies delving into the relationship between hemodynamic and neuronal activities during the resting state. It has been revealed that, although multiple EEG rhythms (i.e. delta, theta, alpha, beta, and gamma rhythms) were all correlated with the hemodynamic fluctuations in one functional network [10], [11], the dominant rhythms were quite different between different functional systems. For example, the brain activities in the attention network were strongly correlated with power changes of alpha rhythm [12], [13], [14], while the activities in the default mode network were more correlated with power changes of the beta rhythm [10], [13]. All the evidence above collectively suggests that the interaction between spatially distinct cerebral activities is not only frequency specific but also functional system dependent. Determination of RSFC_df_ of a functional system is of importance for understanding the brain interaction within the system. In addition, with the increased appreciation of the RSFC investigations in both basic and clinical neurosciences, accurate RSFC_df_ is also essential to assess the extent of RSFC within a specific functional system in order to avoid a biased conclusion.

In recent years, a promising noninvasive imaging technique, functional near-infrared spectroscopy (fNIRS), has been successfully utilized to assess RSFC in a resting-state brain [15], [16], [17], [18], [19], [20], [21], [22]. In assessing RSFC_df_, fNIRS holds distinct merits. First, the high sampling rate of fNIRS avoids aliasing of the high frequency noise to the low frequency spontaneous fluctuations that we are interested in [23], [24], making the determination of RSFC_df_ more accurate. Second, the fNIRS signal represents local brain activity directly below the probes, thus avoid the conductive effect in EEG studies. This property is a desirable property for assessing RSFC_df_ within a specific functional system. Third, the silent environment of the fNIRS scanning excludes the potential confounding led by MRI scanning noise in RSFC_df_ evaluation, not only for auditory related systems, but also for higher functional systems such as attention. Additionally, fNIRS measures three types of hemodynamic parameters (i.e. oxy-Hb, deoxy-Hb, and total-Hb), and thus provides the possibility of understanding the frequency characteristics of RSFC more comprehensively [25]. Finally, fNIRS scanning has fewer constrictions on subjects, allowing fNIRS to study populations not amenable to fMRI, such as infants, young children, the elderly and patients [25], [26], [27].

However, an obstacle that hinders fNIRS from potential RSFC_df_ investigation is the noise interferences. Systemic physiological fluctuations, such as pulsation- and respiration-related fluctuations as well as low frequency vasomotion waves, usually exhibit high covariance across fNIRS measuring channels due to their common vasculature-related origins [26], [28]. Such high spatial covariance may result in an additional synchronization in fNIRS signals between two measurement regions, thus reducing the spatial specificity of the target neuro-related RSFC [15], [16], [19]. Moreover, these systemic physiological fluctuations usually cover a broad frequency band and have complex noise structure in the frequency domain [26], [28], which will degenerate the accuracy of the detection of the dominant frequency. Another artifice in fNIRS data originates from head motion, usually caused by the physical displacement of the optical probe from the scalp. Motion artifact, with large jumps in the fNIRS data, usually occurs in a phase-lock manner across a large measuring area due to the rigid helmet [26], [29]. In addition, the motion-induced large jump usually occurs rapidly and randomly in the whole recording period, resulting in irregular and widely distributed frequency attributes. Though efforts have been made to address this problem, such as fitting the probe to the head as firmly as possible by using a cap or frame and placing optical fibers at right angles to the scalp surface, the motion artifact cannot be completely eliminated, particularly in studies of patients, infants or children. The motion artifact thus may also interfere with the RSFC_df_ analysis due to its relatively wide distribution in spatial domain and complex time-frequency structure.

To solve the problem, a novel conception was initially proposed that functional system specific spatial information can be used to reduce the adverse effect of these global noises on the detection of the dominant frequency of RSFC in the functional system. [24]. In this paper the effect of various global noises on detection of the RSFC_df_ was quantitatively evaluated. Then a functional system dependent and frequency specific analysis method was formally proposed to eliminate these interferences. The validity of this method is proved by using fNIRS data from both simulation and real experiments.

## Theoretical Analysis

The proposed computational framework consists of three basic parts: (1) construction of a spatio-frequency connectivity matrix, (2) spatially weighted coherence analysis, and (3) determination of dominant frequency of RSFC.

### Spatio-frequency Connectivity Matrix

Several metrics have been employed in previous studies of frequency characteristics of RSFC. Some studies used the cross-correlation coefficient at multiple narrow frequency bands [8], [23]; some decomposed correlation coefficient in frequency domain [7], [30]; while other studies adopted a coherence coefficient [23], [31]. In this study, we also used the coherence coefficient in order to setup our computational framework for scoring the frequency characteristics of RSFC. The coherence coefficient quantifies the frequency-specific degree of the time-invariant linear relationship of brain activities (i.e. time series) between two separate spatial units (i.e. brain areas) and is defined as follows [32]:
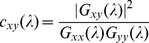
(1)where 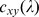 is the coherence coefficient of brain activities between area *x* and *y* at frequency *λ.* 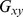 is the cross-spectrum of the brain activities between *x* and *y;* 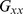 and 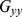, the autospectrum of *x* and *y*, respectively:
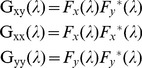
(2)where 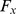 and 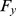 is the Fourier transform of the brain activities at *x* and *y*, respectively, and the asterisk indicates the complex conjugate. The coherence coefficient 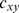 is a function of frequency *λ* and bounded between 0 and 1, where 0 indicates an absence of any linear relationship between *x* and *y*, and 1 indicates that x is perfectly related with y in a linear fashion.

**Figure 1:**
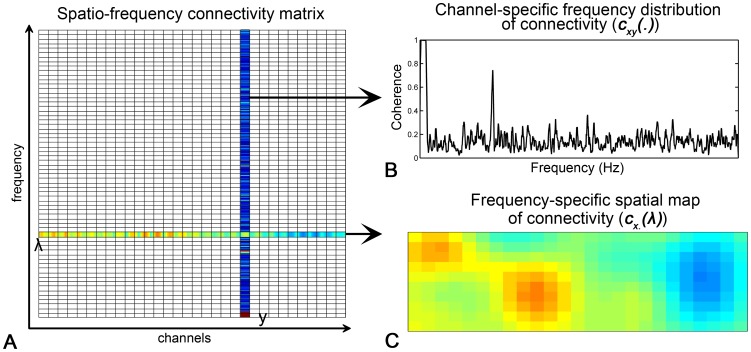
Spatio-frequency connectivity matrix. (A) A schematic diagram of spatio-frequency connectivity matrix, with the horizontal axis labeling all the channels and the vertical axis indexing all the frequencies. (B) Frequency distribution of the connectivity between a channel *y* and the seed channel. It corresponds to one column of the spatio-frequency connectivity matrix. (C) Spatial map of connectivity to the seed channel at a specific frequency point. It is redrawn from one row of the spatio-frequency connectivity matrix.

Given a predefined seed channel *x* and any other channel *y,* the coherence between the two channels at a given frequency *λ* was defined as 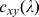 as in (1). For all the channels *y* and frequencies *λ*, a two-dimensional matrix 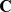 was constructed as illustrated in Fig. 1(A). Each column of 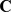 represents a frequency distribution of the connectivity between the seed channel (i.e. *x*) and another channel (i.e. *y*), denoted as 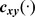. As shown in Fig. 1(B), a higher coherence value at a specific frequency indicates more contribution of this frequency to the connectivity between the two channels. On the other side, each row of matrix 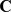 represents a spatial distribution of the connectivity between the seed channel and all other channels at a frequency *λ*, denoted as 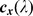. As shown in Fig. 1(C), a higher coherence value at a specific channel suggests higher connectivity between this channel and the seed channel at the given frequency *λ*. Therefore, the matrix 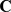 is a spatio-frequency representation of RSFC in nature, denoted as the spatio-frequency connectivity matrix.

### Spatially Weighted Coherence Analysis

In the proposed method, we took advantage of a predefined probability map (i.e., 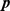) to introduce the spatial information of the interesting functional system (e.g. motor system) and calculate a spatially weighted sum of the coherence coefficient at each frequency as follows:
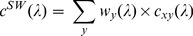
(3)

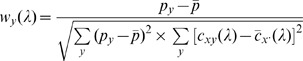
(4)where 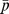 and 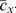 represent the mean value of the map 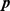 and 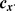 across all the measurement channels 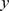, respectively. The 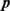 can be defined either functionally (i.e., based on the pattern of task activation) or anatomically (i.e., based on anatomical landmarks) [33], [34]. 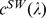 is the spatially weighted coherence coefficient at frequency 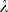 with weight of 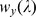. 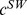 is a natural representation of the similarity between the frequency-specific connectivity map 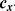 and the template 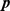 (detailed discussion is provided in the Text S1). A higher value of 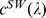 suggests a greater contribution of the spontaneous brain activity to the RSFC in the functional system of interest. In contrast, a lower value suggests insignificant neuro-related RSFC.

For the frequencies which are dominated by the global noises in fNIRS data (e.g., systemic physiological fluctuations and motion artifacts), as discussed in Introduction section, there should be an intense and widespread coherence among all the measurement channels. Such global characteristic of the connectivity map is quite different from the locally distributed template 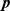, and a low spatially weighted coherence coefficient value can thus be expected. For the frequencies where the global noises and the spontaneous fluctuations are both present dominantly, the connectivity map should be elevated globally due to the noise interference, but the spatial pattern of functional system specific distributions in the map will remain in theory. In this condition, a high 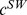
value can be obtained, in line with expectations. As a result, the spatially weighted coherence reduces the noise interference and improves the specificity of the resultant frequency characteristics of RSFC.

### Determination of Dominant Frequency of RSFC

To quantify the dominant frequency of RSFC, we proposed a non-parametric procedure to compute the statistical significant of 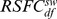 value. First, we built a null distribution of 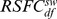 by collecting all the 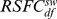 values in the frequencies which were mainly contaminated by the random instrumental noise. Specifically, in consideration of the frequency distribution of the principle physiological noise and the spontaneous brain activity, the ultra-high frequency band (≥3 Hz) were selected to obtain the null distribution in this study. Then, based on the null distribution of 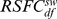, a *p* value was assigned for each 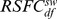 value at all frequencies. Finally, the successive frequencies (more than 3 frequency points) with significant 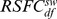 values (p<0.05) was defined to be the dominant frequency of RSFC for the functional system of interest (denoted as 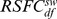).

**Figure 2:**
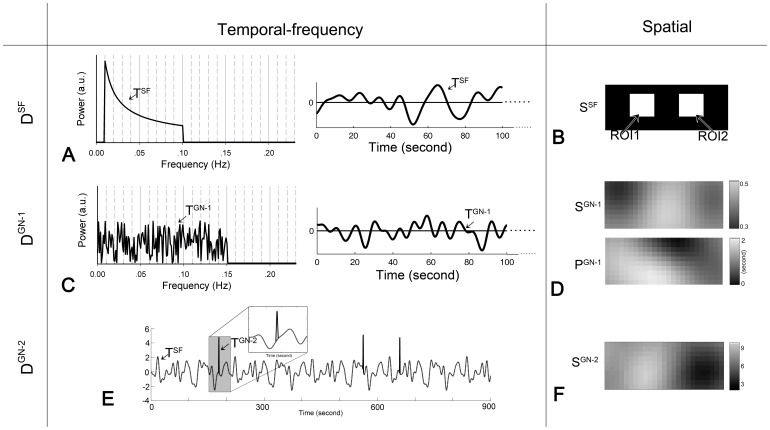
The simulated dataset. (A, C, E) Temporal-frequency characteristics of the simulated dataset. (B, D, F) Spatial characteristics of the simulated dataset. 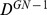 denotes the spontaneous fluctuations, 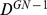 the global noise 1 (i.e., the systemic physiological noises), and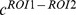 the global noise 2 (i.e., the motion artifact).

To quantity the accuracy of the 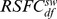 determination, we estimated its specificity and sensitivity in all the following simulative experiments as follows:
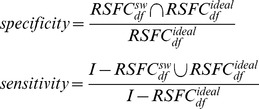
(5)where the ideal frequency band of RSFC (i.e. 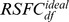) was set as 0.01–0.1 Hz in all the following simulated data sets as shown in Fig. 2(A). *I* was set as 0–0.25 Hz as the whole frequency range in which the specificity and sensitivity were assessed. Furthermore, considering the possible effect of the biased threshold selections on the resultant 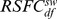, a receiver operator characteristic (ROC) approach was also proposed to further evaluate the specificity and sensitivity of the proposed method [20].

For comparison, we also evaluated the performance of the traditional coherence analysis method. In the traditional coherence method, the connectivity within a certain functional system (e.g., the motor system) is directly represented by the coherence between the averaged time courses from two representative ROIs in the system (e.g. the left and right primary motor areas) [23], [32]. It has no consideration of spatial information to reduce the adverse effect of the global noises.The coherence coefficient (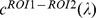) between two ROIs was calculated against the frequency 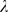 as the formula (1). The dominant frequency of RSFC estimated by the traditional method (denoted as 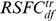) was determined by the same method as for 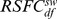.

## Experiments

A series of simulated and real fNIRS experiments were conducted in this study. In general, each set of simulated data 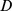 was composed of three basic parts: the spontaneous fluctuations 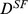, the global noise 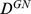, and the instrumental noise 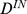 (i.e. 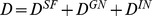).

Specifically, the components of spontaneous fluctuations were generated using the following model:
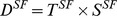
(6)



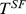
 is the basic temporal profile of the spontaneous fluctuations at low frequencies (0.01–0.1 Hz) [35] (as shown in Fig. 2(A)). It has15 min length with a sampling rate of 10 Hz (9000 time points). 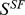 represents the spatial characteristics of the spontaneous fluctuations (as shown in Fig. 2(B)). The sign “×” denotes the exterior product. For additional details, please see the Text S2. The instrumental noise 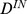 was generated as a spatial-specific Gaussian noise with a signal-to-noise ratio (SNR) of 1 [36]. For the global noise 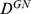, two categories (the systemic physiological noise 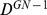 and the motion artifact 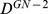) were simulated separately using their own temporal and spatial characteristics as detailed in the following sessions.

### Global Noise 1: Systemic Physiological Noise

To evaluate the potential influence of the systemic physiological noises, such as pulsation- and respiration-related oscillations as well as low frequency vasomotion waves, on the RSFC_df_ determination, a comprehensive systemic physiological noise was simulated at each measuring location *k* as follows:
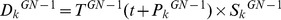
(7)where 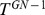 represented the basic temporal profile of the systemic physiological noises as shown in Fig. 2(C). As we know, the systemic physiological noises cover a broad frequency band. Among them, the very-low frequency fluctuations (∼0.04 Hz), Mayer waves (around 0.1 Hz), and respiration-related oscillations (∼0.25 Hz) might overlap with the spontaneous hemodynamic activities in the frequency domain [26], [37], [38]. The pulsation-related fluctuations (∼1 Hz) are, however, believed to be at higher frequency locations without any overlapping. To comprehensively investigate the possible effects of the systemic physiological noises on the frequency characteristics of RSFC, a frequency range of 0∼0.15 Hz for the physiological noises, which is wider than the range for spontaneous fluctuations (0.01∼0.1 Hz), was designed to simulate both, overlapping and non-overlapping, situations between 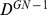
and 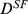. For the spatial characteristics of the systemic physiological noises, although they have highly spatial covariance across measurement channels, they remain different in both phase delay and amplitude across channels [28], [39], [40]. Therefore, complicated spatially global attributes were considered. Two global but spatially inhomogeneous distribution maps (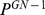 and 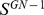) were generated to simulate the relative phase delay and amplitude difference respectively, as shown in Fig. 2(D). For additional details, please see the Text S2. In order to cover the complex situations of the temporal and spatial characteristics of the systemic physiological noises encountered in real circumstances, the simulated data sets were randomly generated 100 times.

### Global Noise 2: Motion Artifact

To assess the influences of motion artifact on the RSFC_df_ determination, the motion artifact 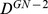 was generated as follows:
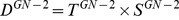
(8)where 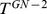 was the basic temporal profile of motion artifacts generated with a temporal random occurrence as shown in Fig. 2(E). 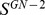 was the spatial distribution of the amplitude of the motion artifacts which may be different across channels due to the different curvature of the probe at different head positions, as shown in Fig. 2(F) [41]. For additional details, please see the Text S2. Also, the 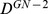 was randomly generated 100 times considering the random nature and individual variability of the motion artifacts.

### Template

In the proposed method, the spatial template 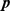 plays a key role in eliminating the adverse effect of the global noises. Thus it is important to evaluate the robustness of the method to the possibly over- or under-estimated size and shifted location of the functional system of interest (represented as 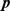). In order to simulate the biased estimation of size, the simulated functional system indicated as the two ROIs in Fig. 2(B) was expanded (shrunk) 0%, 10% and 20% of the original size respectively. As for the biased estimation of location, the simulated functional system was shifted horizontally from the original location 0%, 10% and 20% respectively. For each case, the 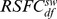 was calculated, and the influence was evaluated.

### Real Resting-state fNIRS Experiment

Twenty-one subjects were recruited from Beijing Normal University. Informed consent was obtained before the experiment according to the procedure approved by the Review Board at State Key Laboratory of Cognitive Neuroscience and Learning, Beijing Normal University. All subjects participated in two sessions of fNIRS measurements. The former was 11-min resting state session, and the latter was a sequential bilateral finger tapping task (see detailed descriptions in [21], [22]). The fNIRS measurements were conducted with a 52-channel ETG-4000 Optical Topography System (Hitachi Medical Co., Tokyo, Japan) at a sampling rate of 10 Hz. The 17 emitter and 16 detector optodes were plugged into a holder and covered the bilateral sensorimotor areas according to the international 10–20 system [42]. Sixteen subjects were involved in this study according to the exclusion standard in our previous study (right-handed and rescanned after one week) [21], [22]. The concentration changes of HbO signal were computed with the modified Beer–Lambert law [43]. For the localizer task data, the data were high-pass filtered (>1/60 Hz) after discarded the first 30 s data. The group-level task activation map (*t*-map) was obtained based on the general linear model [16] and the channel with the greatest activation (ch24) was chosen as the seed-channel. For the resting state data, the first 20 s and the last 40 s signals were discarded due to unstable. The proposed computational framework was applied to the remained 10 min data to assess the dominant frequency of RSFC within the sensorimotor area. The anatomical localization of the sensorimotor area (Fig. 2(B) in [21]) was used as the spatial template after it was smoothed by a 3-by-3 kernel matrix in order to reduce the anatomical variability across subjects [44].

## Results

### Global Noise 1: Systemic Physiological Noise

**Figure 3:**
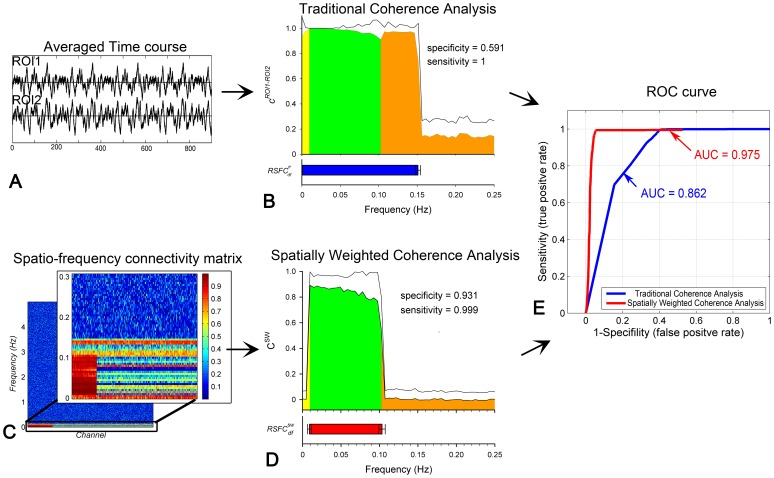
RSFC_df_ results of the simulated experiment with contamination of the systemic physiological noise. (A) An example of the average time course from the two ROIs (shown in Fig. 2B) for the traditional coherence analysis. (B) The mean coherence curve between the two ROIs (i.e., 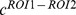) over 100 times of stimulations derived from the traditional method. The coherences at frequency >0.25 Hz were not plotted because of very low values. The black line represents the standard deviation. The green area indicates the 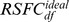 (i.e., 0.01∼0.1 Hz), and the yellow and the orange area indicate the frequencies lower or higher than 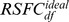, respectively. The blue horizontal bar indicates the 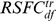. (C) An example of the spatio-frequency connectivity matrix (bottom layer) and an enlargement over the low frequency portion below 0.3 Hz (top layer). (D) The mean spatially weighted coherence curve over 100 times of stimulations derived from the proposed method (i.e., 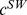). The black line represents the standard deviation. The red horizontal bar indicates the estimated 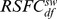. (E) ROC curves for RSFC_df_ determination of the two methods with blue for the traditional method and red for the proposed.

In the first simulative experiment, the data was generated as 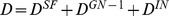 and was used to evaluate the possible influence of the systemic physiological noise on the RSFC_df_ determination. The results from both the traditional coherence method and the proposed one are shown in Fig. 3. For the traditional method, the two averaged time courses (shown in Fig. 3(A)) are calculated from the two ROIs in the simulated functional system (shown in Fig. 2(B)) respectively, and the coherence between them (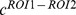) represents the frequency-specific RSFC of the corresponding functional system. The averaged 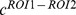 curve over 100 times of random simulations is plotted in Fig. 3(B) with the standard deviation represented as the black line. The coherence at frequency >0.25 Hz were not plotted because of very low values. The resultant 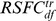 (the blue horizontal bar) is located from 0.0001±0.0008 Hz to 0.1510±0.0032 Hz (mean±std), manifestly beyond the ideal 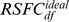 (0.01–0.1 Hz, marked as the green area) at both the lower (marked as the yellow area) and higher (the orange) frequencies. This is nearly identical to the primary frequency band of the simulated systemic physiological noises (0–0.15 Hz, shown in Fig. 2(C)). The specificity of 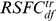 is 0.591±0.020 (mean±std), and the sensitivity is 1 for all the 100 times of random simulations. This reveals a significant disturbance of the systemic physiological noises to the RSFC_df_ determination by using the traditional method. The spatio-frequency connectivity matrix of the seed channel (the central pixel of the ROI1 in Fig. 2(B)) as well as an enlargement of its low-frequency portion is shown in Fig. 3(C). On this basis, along with the spatial template (shown in Fig. 2(B)), the spatially weighted coherence (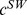) is computed and plotted in Fig. 3(D) where the resultant 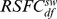 is represented by a red horizontal bar. The 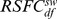, from 0.0090±0.0026 to 0.1033±0.0041 Hz, was more proximal to the ideal 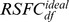 than the 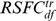. Its specificity is 0.931±0.026 (mean±std), much higher than 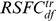, while the sensitivity is equivalent to 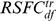 (0.999±0.011). It indicates that the proposed method successfully eliminates the adverse effect of the globally distributed systemic physiological noises. Moreover, as shown in Fig. 3(E), the proposed method (red line, the area under the ROC curve [AUC] = 0.975) significantly outperforms the traditional one (blue line, AUC = 0.862). This result further confirmed that the proposed method has higher specificity as well as sensitivity than that of the traditional one while identifying the RSFC_df_.

### Global Noise 2: Motion Artifact

**Figure 4:**
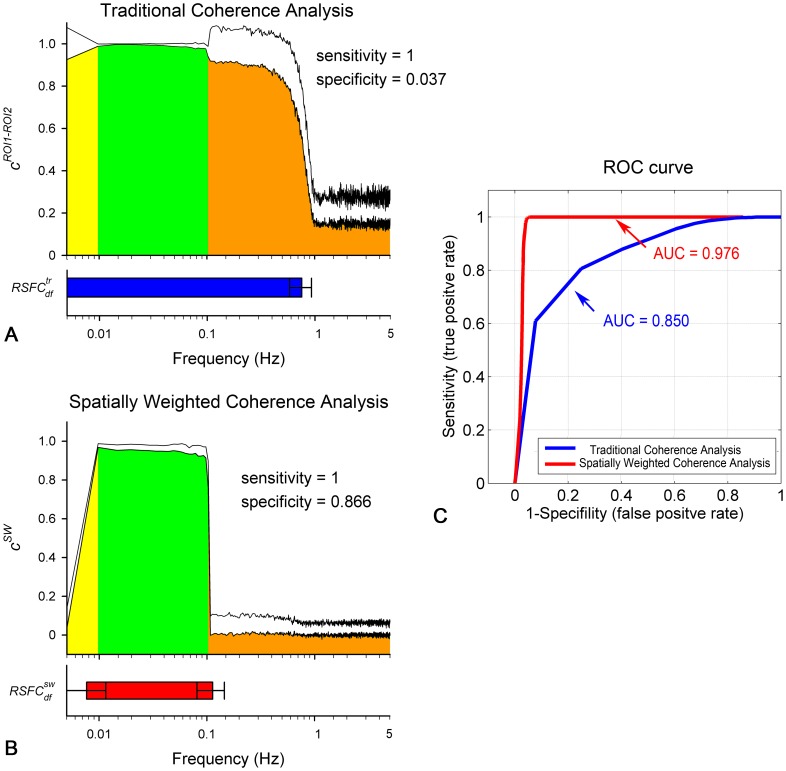
RSFC_df_ results of the simulated experiment with contamination of the motion artifact. (A) Semilog plot of the mean coherence curve between the two ROIs over 100 times of stimulations derived from the traditional method (i.e., 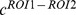). The black line represents the standard deviation. The green area indicates the 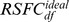 (i.e., 0.01∼0.1 Hz), and the yellow and the orange area indicate the frequencies lower or higher than 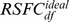, respectively. The blue horizontal bar indicates the 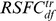. (B) Semilog plot of the mean spatially weighted coherence curve over 100 times of stimulations derived from the proposed method (i.e., 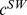). The black line represents the standard deviation. The red horizontal bar indicates the estimated 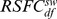. (C) ROC curves for RSFC_df_ determination of the two methods with blue for the traditional method and red for the proposed.

In the second simulative experiment, the data was generated as 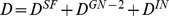 in order to investigate the influence of the motion artifact. The results from both of the traditional and the proposed methods are shown in Fig. 4. As shown in Fig. 4(A), the resultant 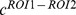, drawn on a semilogarithmic graph, are significant over a quite broad frequency range. The corresponding 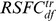 (blue horizontal bar) is located from 0.0002±0.0014 Hz to 0.754±0.173 Hz which is much broader than the ideal 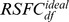 (0.01∼0.1 Hz, marked as the green area). The specificity of the traditional method to the dominant frequency of RSFC is 0.037±0.178 (mean±std), although the sensitivity is 1. Further power spectral analysis of the motion artifact shows that the primary frequency components of the artifacts are also located in the same frequency band as 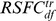, demonstrating a heavy influence of the global artifacts on RSFC_df_ determination by using the traditional method. In contrast, as shown in Fig. 4(B), the 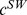 from the proposed method are concentrated over a low frequency range on a semilogarithmic graph. The 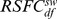 is located from 0.0077±0.0039 Hz to 0.113±0.032 Hz. Its specificity is 0.866±0.197 (mean±std), much higher than 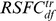, while the sensitivity is equivalent to 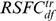 (equal to 1 for all the 100 times of random simulations). The ROC results further validated that the proposed method (AUC = 0.976) outperforms the traditional one (AUC = 0.850) (shown in Fig. 4(C)). All these results show that the proposed method can effectively reduce the influence of the global artifact and thus achieve more accurate estimation of the frequency band of RSFC.

### Template

**Figure 5:**
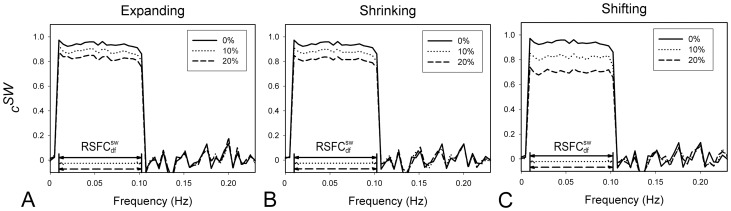
The robustness of the proposed method to the possible inaccurate spatial template. (A) The 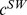 results with the expanded spatial template in which the size of the functional system of interest is overestimated. (B) The 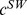 results with the shrunk spatial template in which the size of the functional system of interest is underestimated. (C) The 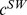 results with the spatial template which inaccurate estimate the location of the functional system of interest. The dotted line represents the case of template with 10% estimated errors. The dashed line indicates the case of template with 20% estimated errors. And the solid line is for the original template without errors. The horizontal arrow indicates the estimated 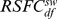 range for each case.

The robustness of the proposed method to the possible inaccurate predefined spatial template was evaluated and the results are shown in Fig. 5. The resultant 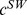 for the over- or under-estimation of the functional system’s size are plotted in Fig. 5(A) and Fig. 5(B) respectively. Although the amplitudes of 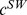 are slightly decreased, the 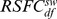 is almost identical to the ideal situation (0% deviation) with 10% and 20% deviation in size. These results indicate that moderate deviation of the spatial template has little impact on the final identification of the 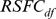. The resultant 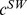 for inaccurate estimation of the system’s location is plotted in Fig. 5(C). Similar to the case of inaccurate size of the template, the 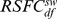 for different deviations (10% and 20%) in position is almost consistent with those without deviation. Overall, the experimental results indicate the robustness of our method to the over- or under-estimation of the size and location of the functional system of interest represented by the spatial template.

### Real Resting-state fNIRS Experiment

**Figure 6:**
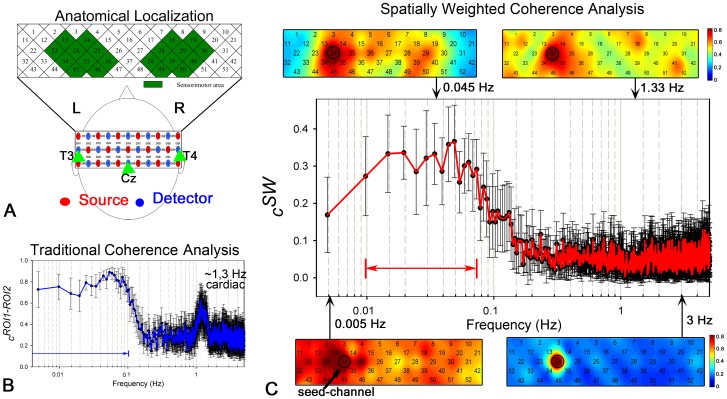
Comparison between the proposed and traditional methods on the real resting-state fNIRS dataset. (A) Schematics of the fNIRS probe array and the anatomical localizations of the fNIRS channels. The labeling maps with green indicate the channels covering the sensorimotor area. (B) The coherence curve of the bilateral sensorimotor areas across all the subjects derived from the traditional method. The error bar indicates the standard error. The horizontal blue arrow at the bottom indicates the estimated 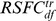. (C) The group results of the proposed method with four typical frequency-specific connectivity maps. The error bar indicates the standard error across subjects, and the horizontal red arrow indicates the estimated 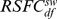.

For the real resting-state fNIRS experiment in the sensorimotor area, a schematic diagram of the probe location is shown in Fig. 6(A). By using the traditional method, the 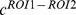 curve between the bilateral sensorimotor areas (the left and right region in green in Fig. 6(A)) is calculated for all the subjects. The averaged 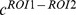 curve across all the subjects is plotted on a semilogarithmic graph as shown in Fig. 6(B), with the error bar indicating the standard error across subjects. It is obvious that, the 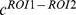 is greater in the low frequencies (0∼0.1025 Hz, 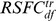, indicated by the blue arrow) and the pulsation-related frequencies (∼1.3 Hz). For comparison, the averaged 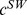 curve across subjects by using the spatially weighted coherence analysis is plotted in Fig. 6(C). As opposed to the dominant frequency band of 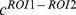, the dominant frequency of the 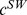 (i.e., 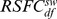, indicated by the red arrow) is concentrated between 0.01 Hz and 0.0732 Hz. The significantly decreased connectivity at ultra-low frequency (<0.01 Hz), low frequency (∼0.1 Hz, the Mayer’s wave), and the pulsation-related frequency (∼1.3 Hz) might suggests that the proposed method eliminates the various global noise effectively.

Four typical frequency-specific connectivity maps are shown in Fig. 6(C) in order to further demonstrate why the proposed method can reduce the global noise disturbance to the RSFC_df_ determination. In particular, the map in the top left, specific to the frequency of 0.045 Hz, represents a symmetrical connectivity pattern covering the bilateral sensorimotor areas which resembles the anatomical location of the sensorimotor area (Fig. 6(A)). This amazing spatial pattern, corresponding to a great 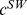 value, suggests the great potential of the frequency of 0.045 Hz to contribute to the synchronization of the neuro-related spontaneous fluctuations. Conversely, the maps in the top right and the bottom left with a very low 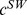 values, specific to the cardiac frequency of 1.3 Hz and the ultra-low frequency of 0.005 Hz, respectively, shows high global connectivity. This phenomenon suggests that the fluctuations at these frequencies mainly consist of several globally distributed interferences not related to the neural activities. At a ultra-high frequency (3 Hz, arbitrarily selected), the map (the bottom right one) with a very low 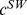 value did not show any meaningful connectivity, suggesting an instrumental noise at this frequency.

## Discussion and Conclusions

In this study, we provided a comprehensive assessment of the interference of the global noise to the determination of the dominant frequency of RSFC and proposed a functional system dependent and frequency specific analysis method to eliminate the adverse influences of the interference. To evaluate the performance of the proposed method, we simulated the spontaneous fluctuations contaminated by the two main types of noise which are globally distributed in fNIRS dataset (i.e., the systemic physiological interferences and the motion artifacts). The simulations were close to the conditions in the real fNIRS experiment at our utmost. The simulated experimental results clearly showed that all types of global noises generated redundant correlations in the frequency band unrelated to spontaneous neural activity and resulted in a low specificity of RSFC_df_ results when spatial information was not considered. This unfavorable phenomenon hints that the RSFC_df_ results derived from the traditional method should be interpreted with caution [23], [45]. On the contrary, the proposed method greatly eliminated the adverse effects of both of the systemic physiological interferences and the motion artifacts from the frequency characteristics of RSFC and yielded RSFC_df_ results that were more specific to the spontaneous neural activities. Such improvement is significant even in a worse scenario (e.g. the amplitudes of the systemic physiological noise were two or three times higher than the spontaneous one [46], which is not shown in the text). Furthermore, based on the real resting-state fNIRS dataset, the connectivity in the sensorimotor area derived from the proposed method was concentrated at the low frequencies (0.01∼0.0732 Hz), whereas the results from the traditional method was widely distributed and included several typical physiological noise frequencies. This result further validates the ability of the proposed method to improve the determination of the RSFC_df_.

In previous fNIRS-based RSFC studies, various methods have been utilized for the general purpose of noise removal; however none of the methods were intensive enough to address the problem of the global interference mentioned above. A simple and widely adopted approach is temporal filtering [16], [19], [23], [45]. This method removes noise components with ultra-low- and/or high-frequency spectra from the measured signals, such as the long-term drift and the cardiac pulsations. However, for noise components overlapped with the neural activity related spontaneous fluctuations in frequency domain (e.g., the Mayer’s wave, around 0.1 Hz), band-pass filtering usually shows little effectiveness [26]. Another approach is using additionally recorded noise as a reference and removing it from the measurement data with a linear regression. Such reference usually is the signal derived either from the short-separation emitter–detector pairs [15] or from the auxiliary instruments [47]. Both theoretical and practical considerations limited this method in the fNIRS-based RSFC_df_ studies. On one hand, the underpinning theoretical assumption of this method, that the observed hemodynamic signal can be expressed as a linear sum of the spontaneous fluctuations and the systemic physiological terms, is not clearly proved [48]. On the other hand, this method requires a specially designed emitter–detector arrangement with multiple separations or additional physiological signal recoding systems. This is, however, not always available in all existing fNIRS devices. Independent component analysis (ICA), as a powerful blind source separation method, has also been used to separate noise components and identify RSFC of multiple function systems from resting-state fNIRS measurements [20], [21]. However, more experimental evidence is desired to demonstrate the ability of ICA to separate all specimens of global noise and artifacts which may interfere with the fNIRS-based RSFC_df_ analysis.

As demonstrated by theoretical analysis, the predefined spatial template of the functional system of interest is an important parameter in the proposed method. Thus, its accuracy may have influence on the performance of the proposed method. To assess this potential impact quantitatively, we simulated the template with different levels of position or/and size discrepancy to represent the inaccuracy of the estimated template in the actual experiment. The results showed that the method was robust to the discrepancy of the template at a moderate degree. Furthermore, in the real resting-state fNIRS experiment, we tested two different template generation approaches. The first template was constructed according to the anatomical localization information of each channel, and the second one was made from the task activated result during the motor task (*p*<0.05 at group-level, FDR corrected, not presented in the text). Despite slight differences in the two templates, the outputs of the proposed method were quite consistent. Moreover, the spatial template can be appropriately smoothed to further reduce the influence of template estimation error on the final results.

Despite some meaningful findings on the RSFC_df_ were found by the previous studies, the preliminary results are still inconsistently. For example, Salvador et al. observed that the RSFC_df_ in the primary auditory area was over a broad frequency range (0∼0.25 Hz), and in the occipital cortex, especially in primary visual and related regions (calcarine and lingual cortices), was at both low (<0.08 Hz) and high (0.17–0.25 Hz) frequencies [9]. However, in other studies, the RSFC in the auditory and visual area was only concentrated in low frequencies below 0.1 Hz or even below 0.05 Hz [7], [8], [30]. Even for the same “low-frequency” RSFC, the precise low-frequency band of RSFC concentration still have inconsistencies. Taking the sensorimotor area as an example; Cordes et al., found RSFC were most concentrated in the frequencies lower than 0.05 Hz in the early study [30], whereas in their later publication the dominant frequency was broader over 0–0.1 Hz [7]. Similar differences also appeared in two articles by Beckmann [49], [50]. Therefore, significant effort is still required to fully clarify RSFC_df_ in basic and clinical neuroscience. The discrepancies of the preliminary results may have resulted from the slight influences of the strident noise and the direct visual stimulation in the fMRI scanner, and/or an aliasing effect due to the low sampling rate of fMRI; however, the introduction of fNIRS into RSFC_df_ studies can basically overcome these issues. Moreover, because of the portable and cost-effective features of fNIRS, along with the plainness of the proposed method in this study, the RSFC_df_ investigation can be largely carried out for both of healthy populations and patients with neurologic and psychiatric disorders in the future.

The proposed method also has potential to be adapted for assessing fMRI-based RSFC_df_. Similarly to the fNIRS-based RSFC_df_ analyses, fMRI studies also have suffered from the global distributed physiological interference and motion artifacts [9], [51], [52], [53]. To eliminate these confounds, earlier studies proceeded with much caution by using a temporal independence component analysis or improving sampling rate of fMRI [7], [8]. It should be noted that the spatial behavior of desired system-specific fMRI-based RSFC and global interference is distinct. It coincides with the basic hypothesis underlying the proposed method in this study, suggesting that the proposed method should have potential to be adapted for fMRI-based RSFC_df_ study. Further work should be carried out to test its feasibility in practical.

Despite the merits mentioned above, it is necessary to point out that the proposed method is effective in the case of the large brain area measurement. To construct the spatial template, the spatial coverage of the probe should comprise both inside and outside of the functional area of interest. If there is no sufficient spatial coverage (e.g. only covering a small number of interested channels), the performance of the proposed method will be limited. Fortunately, as the multi-channel measurement fNIRS technology is becoming mature and popular in recent years [23], [28], [47], this problem is not a grave restriction in practice. In addition, although the prior anatomical template plays a key role in elimination of the adverse effect of the global interference, it did limit the application of this method at a system level. That is, if there were adequate information to get the spatial templates of multiple functional systems, it could be applied to explore the frequency system in which RSFC_df_ has changed during development, normal aging, or due to neurological or psychiatric illness. However, it seems to be precluded from more complicated situations, such as investigating the frequency of RSFC between different functional systems (e.g. between the frontal area and the occipital area) [9], [17], [23], and exploring the RSFCdf at voxel and/or region level.

## Supporting Information

Text S1Supplemental Information on 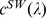.(DOC)

Text S2 Data Generation for Simulative Experiments.
(DOC)
