# Supplementary material for: Determination of Dominant Frequency of Resting-State Brain Interaction within One Functional System
Source: PLoS One. 2012 Dec 17;7(12):e51584. doi: 10.1371/journal.pone.0051584 (PMC3524243; doi:10.1371/journal.pone.0051584)
Supplement: Text S1 — Supplemental Information on . (DOC) [file pone.0051584.s001.doc]

**Text S1: Supplemental information on** $c^{SW}(\lambda)$

We defined $c^{SW}(\lambda)$ as below to assess the similarity of the spatial map of connectivity (i.e. $c_{x\cdot}(\lambda)$) to the prior spatial probability template of the functional system of interest (i.e. $p$) at each frequency.

$\begin{matrix} c^{SW}(\lambda)=r(\lambda) =\frac{\sum_{y} (p_{y}-\bar{p})\times[c_{xy}(\lambda)-\bar{c}_{x\cdot}(\lambda)]}{\sqrt{\sum_{y} (p_{y}-\bar{p})^{2}\times\sum_{y} [c_{xy}(\lambda)-\bar{c}_{x\cdot}(\lambda)]^{2}}} \\ =\frac{\sum_{y} (p_{y}-\bar{p})\times c_{xy}(\lambda)}{\sqrt{\sum_{y} (p_{y}-\bar{p})^{2}\times\sum_{y} [c_{xy}(\lambda)-\bar{c}_{x\cdot}(\lambda)]^{2}}}-\frac{\bar{c}_{x\cdot}(\lambda)\times\sum_{y} (p_{y}-\bar{p})}{\sqrt{\sum_{y} (p_{y}-\bar{p})^{2}\times\sum_{y} [c_{xy}(\lambda)-\bar{c}_{x\cdot}(\lambda)]^{2}}} \end{matrix}$With , we have

$$\begin{matrix} c^{SW}(\lambda) =\frac{\sum_{y} (p_{y}-\bar{p})\times c_{xy}(\lambda)}{\sqrt{\sum_{y} (p_{y}-\bar{p})^{2}\times\sum_{y} [c_{xy}(\lambda)-\bar{c}_{x\cdot}(\lambda)]^{2}}} \\ =\sum_{y} \frac{p_{y}-\bar{p}}{\sqrt{\sum_{y} (p_{y}-\bar{p})^{2}\times\sum_{y} [c_{xy}(\lambda)-\bar{c}_{x\cdot}(\lambda)]^{2}}}\times c_{xy}(\lambda) \end{matrix}$$

Thus, we reach a simple expression,

$$c^{SW}(\lambda) =\sum_{y} w_{y}(\lambda)\times c_{xy}(\lambda)$$

As a result, $c^{SW}$ is the weighted sum of the connectivity map with a spatially weighting coefficient of $w$. Accordingly, the $c^{SW}(\lambda)$ is denoted as “spatially weighted coherence coefficient”.
