# Supplementary material for: Determination of Dominant Frequency of Resting-State Brain Interaction within One Functional System
Source: PLoS One. 2012 Dec 17;7(12):e51584. doi: 10.1371/journal.pone.0051584 (PMC3524243; doi:10.1371/journal.pone.0051584)
Supplement: Text S2 — Data Generation for Simulative Experiments. (DOC) [file pone.0051584.s002.doc]

**Text S2: Data Generation for Simulative Experiments**

The basic temporal profile of the spontaneous fluctuations, $T^{SF}$, was designed to follow a spectral distribution of 0.69*1/*f* [1] and implemented as a linear combination of random-phased sinusoids at low frequencies (0.01 - 0.1 Hz) [2]. The spatial characteristic of the spontaneous fluctuations, $S^{SF}$, was designed as a 10-by-25 binary map with two rectangle ROIs indicating the brain functional system where the spontaneous fluctuations originated. The simulated spontaneous fluctuation, at measuring location *k*, is thus the amplitude-modulated basic temporal profile $D_{k}^{SF}=T^{SF}\times S_{k}^{SF}$.

The basic temporal profile of the systemic physiological noises, $T^{GN-1}$, was generated as a linear combination of amplitude- and phase-random sinusoids of 0 ~ 0.15 Hz. The spatial characteristics of the phase delay of the systemic physiological noises, $P^{GN-1}$, was generated as a white Gaussian noise spatial map smoothed by a Gaussian kernel (FWHM = 10) and scaled between 0 to 2 second [3]. The spatial characteristics of the amplitude of the systemic physiological noises, $S^{GN-1}$, was generated in the same way as $P^{GN-1}$ but with the amplitudes between 1/3 and 1/2 times of that of the spontaneous fluctuations [4,5]. Eventually, at a location *k*, the physiological noise $D_{k}^{GN-1}$ is the basic time course $T^{GN-1}$ phase-delayed by $P_{k}^{GN-1}$ and amplitude-modulated by $S_{k}^{GN-1}$.

The basic temporal profile of motion artifacts, $T^{GN-2}$, was generated with a temporal random occurrence. A wider range of occurrence frequency (1-12 spikes during the simulated 15-min recording period) was used to cover the general occurrence frequency for various subjects or populations in real situations [6]. The spatial distribution of the amplitude of the motion artifacts, $S^{GN-2}$, was generated in the same way as $S^{GN-1}$ but scaled from 3 to 10 times of the amplitude of the spontaneous fluctuations [7,8].

**Reference:**

1. He BJ, Zempel JM, Snyder AZ, Raichle ME (2010) The temporal structures and functional significance of scale-free brain activity. Neuron 66: 353-369.

2. Fox MD, Raichle ME (2007) Spontaneous fluctuations in brain activity observed with functional magnetic resonance imaging. Nat Rev Neurosci 8: 700-711.

3. Franceschini MA, Joseph DK, Huppert TJ, Diamond SG, Boas DA (2006) Diffuse optical imaging of the whole head. J Biomed Opt 11: 054007.

4. White BR, Snyder AZ, Cohen AL, Petersen SE, Raichle ME, et al. (2009) Resting-state functional connectivity in the human brain revealed with diffuse optical tomography. Neuroimage 47: 148-156.

5. Katura T, Tanaka N, Obata A, Sato H, Maki A (2006) Quantitative evaluation of interrelations between spontaneous low-frequency oscillations in cerebral hemodynamics and systemic cardiovascular dynamics. Neuroimage 31: 1592-1600.

6. Sato H, Tanaka N, Uchida M, Hirabayashi Y, Kanai M, et al. (2006) Wavelet analysis for detecting body-movement artifacts in optical topography signals. Neuroimage 33: 580-587.

7. Izzetoglu M, Chitrapu P, Bunce S, Onaral B (2010) Motion artifact cancellation in NIR spectroscopy using discrete Kalman filtering. Biomed Eng Online 9: 16.

8. Izzetoglu M, Devaraj A, Bunce S, Onaral B (2005) Motion artifact cancellation in NIR spectroscopy using Wiener filtering. IEEE Trans Biomed Eng 52: 934-938.
